# Responses of Invasive Plants from Different Families to Warming and Drought

**DOI:** 10.3390/plants15071018

**Published:** 2026-03-26

**Authors:** Yu Zhang, Yu Tian, Xiaochen Zhao

**Affiliations:** 1 Key Laboratory of Tree and Grass Geneticsand Breeding, College of Forestry and Grassland Science, Jilin Agricultural University, Changchun 130118, China; zhangyu08240318@163.com; 2 Shenzhen Institute of Standards and Technology, Shenzhen 518033, China; zhaoxiaochen@sist.org.cn

**Keywords:** climate change, invasive species, root traits, synergistic effects

## Abstract

Climate warming and drought often co-occur to form warm–dry climate patterns. However, systematic comparative studies of the responses of invasive plants from different families to their combined effects remain limited. We conducted a greenhouse experiment to investigate the interactive effects of warm (normal vs. warming) and drought (well-watered vs. drought) conditions on the growth, root traits, and competitive performance of 11 invasive plant species from three families (Amaranthaceae, Poaceae, and Asteraceae) growing in competition with native communities. Our results showed that warming did not significantly increase the total biomass of all invasive species combined but significantly promoted biomass accumulation in Poaceae and Asteraceae. Drought consistently reduced the biomass across all invasive species. Notably, a marginally significant interaction effect of warm and drought conditions on the biomass proportion of Amaranthaceae was detected. Specifically, under normal conditions, drought increased the biomass proportion of Amaranthaceae species, whereas under the warming treatment, drought decreased it. Furthermore, root traits of invasive species exhibited clear family level differentiation. Poaceae adopted an expansion strategy by increasing root length and root surface area under warming treatment, Amaranthaceae exhibited a contraction strategy by reducing root investment under drought treatment, and Asteraceae displayed an efficient strategy with increased specific root length under drought treatment. Except for the biomass proportion of Amaranthaceae, no significant interactive effects were found for most other parameters, indicating that the combined effects of warming and drought were primarily additive. Our results revealed that warm, dry climates influence invasive plants in a taxon-specific manner, with different families employing distinct root trait adjustment strategies in response to environmental stress. These findings highlight the importance of family level comparative studies for predicting invasion dynamics and developing targeted management strategies for future climate scenarios.

## 1. Introduction

Alien plant invasions pose a serious threat to biodiversity, ecosystem function, and socioeconomic development [1,2]. Through mechanisms such as resource competition, habitat modification, and allelopathy, invasive plants often lead to a decline in native species, community homogenization, and the loss of key ecosystem services [3,4,5]. In the context of global climate change, increasing temperatures and altered precipitation regimes (e.g., more frequent drought events) are emerging as key drivers of species distribution and interspecific relationships [6,7]. These changes may significantly alter the invasiveness of alien plants and their competitive outcomes with native communities, thereby complicating the prediction and management of invasion risk.

Empirical studies have confirmed that climate change plays a significant role in modulating alien plant invasions [8,9]. Warming can directly promote the physiological metabolism and growth of many invasive species by increasing photosynthetic enzyme activity, extending the effective growing season, and enhancing biomass accumulation, thereby strengthening competitive abilities [10,11]. Furthermore, warming may indirectly facilitate invasion by accelerating soil organic matter decomposition and nutrient mineralization, providing advantages to invasive plants with strong resource acquisition capabilities [12,13]. Altered precipitation regimes are also critical factors affecting alien plant invasions [14,15,16]. Drought stress imposes a universal growth limitation. While it is often assumed that drought inhibits invasive species more strongly than native species, accumulating evidence suggests otherwise. Several studies have found that invasive species can maintain or even enhance their competitive advantage over native species under drought conditions, leading to a further increase in invasiveness [17,18,19]. This is attributed to a suite of adaptive traits commonly exhibited by invasive plants, such as higher water-use efficiency, deeper root distribution, stronger osmotic adjustment capacity, and faster stress recovery, which enable them to maintain relatively high fitness and outcompete native species when water is limited [20,21]. However, warming and drought often co-occur in natural climate systems, forming an increasingly prevalent warm–dry climate pattern [22,23]. The combined effects of these two factors are not merely additive, but may generate complex nonlinear interactions, resulting in unique regulatory effects on plant invasiveness that differ from those of single factors.

Warm–dry climates not only directly affect plant physiological and ecological processes but may also reshape community structure by altering competitive interactions and resource-use efficiency among species [23]. For example, warming may exacerbate soil water evaporation and amplify the physiological stress caused by drought [24]. However, invasive species equipped with efficient water acquisition and utilization strategies may transform this dual stress into a relative competitive advantage [25,26]. Moreover, the synergistic effects of warming and drought can jointly influence the establishment success of invasive individuals and the resistance stability of native communities by modifying soil microbial community functions and nutrient cycling processes (e.g., nitrogen and phosphorus) [6,27,28]. These multifaceted effects indicate that the responses of plant invasion to warm–dry climates may be species-specific and closely linked to the functional traits of the invaders.

Alien plants from different families, owing to their inherent phylogenetic backgrounds and resource-use strategies, may exhibit markedly divergent response patterns to combined warm–dry stress. For instance, Poaceae species typically possess dense fibrous root systems, which may confer a strong capacity for soil water and nutrient capture [29]. Amaranthaceae species often employ the C4 carbon fixation (C4) photosynthetic pathway, potentially providing photosynthetic advantages under high temperatures and water stress [30], and Asteraceae species frequently feature taproots and allelopathic potential, making them prominent in resource competition and disturbed habitats [31,32]. However, current research on the combined effects of warming and drought has largely focused on case studies of a single or a few species, lacking systematic comparative investigations of the response patterns of invasive plants at the family level. Such a cross-family comparative perspective is crucial for revealing the general rules underlying invasion success under warm–dry scenarios and for identifying high-risk invasive plant groups.

To address the research gaps outlined above, we conducted a multispecies greenhouse experiment. We selected 11 invasive plant species and 10 native plant species as the species pool to form the native communities. The native species selected for this experiment are widely distributed across China and frequently co-occur with multiple invasive plants in disturbed habitats. By measuring the aboveground and belowground biomass of invasive plants, as well as root functional traits (including specific root length, root length, and root surface area), we tested the following hypotheses: (1) warming and drought exert interactive effects on plant growth, with their combined effects being non-additive rather than simply additive; (2) invasive species from different families exhibit divergent responses to warming and drought, reflecting family-level differences in adaptive strategies; and (3) under combined warm–dry stress, invasive species maintain or enhance their competitive advantage over native species, contributing to increased invasiveness. By integrating multi-species comparisons with family-level analyses, this study sought to elucidate, from a phylogenetic perspective, the key mechanisms by which future warm–dry climates influence the invasiveness of alien plants, thereby providing a scientific basis for developing taxon-specific invasion risk early warning and management strategies.

## 2. Results

### 2.1. Final Biomass

**Figure 1:**
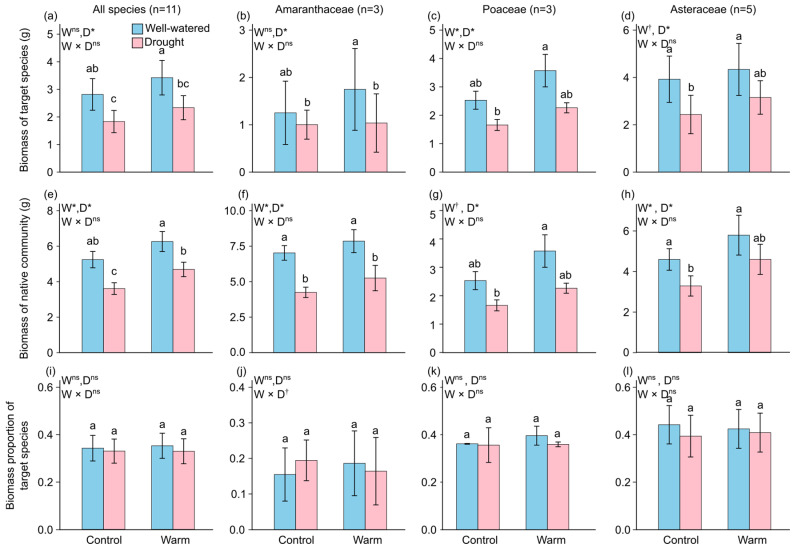
Modeled mean values (±SE) of the biomass of target species for (**a**) all species, (**b**) Amaranthaceae, (**c**) Poaceae, and (**d**) Asteraceae; the biomass of native communities for (**e**) all species, (**f**) Amaranthaceae, (**g**) Poaceae, and (**h**) Asteraceae; and the biomass proportion of target species for (**i**) all species, (**j**) Amaranthaceae, (**k**) Poaceae, and (**l**) Asteraceae under each combination of two warming (normal vs. warming) and two drought (well-watered vs. drought) treatments. Model terms (W, Warm; D, Drought) whose effects are significant (*p* < 0.05) are indicated with asterisks (*), and those whose effects are marginally significant (0.05 < *p* < 0.1) are indicated with daggers (†), ns indicates non-significant (*p* ≥ 0.1). Different lowercase letters above the bars indicate significant differences (*p* < 0.05) between treatment combinations based on post hoc pairwise comparisons of estimated marginal means (emmeans). The four treatment combinations consisted of two levels of warming (normal vs. warming) and two levels of drought (well-watered vs. drought).

On average, when all target species were analyzed together, warming did not significantly increase overall biomass. However, it significantly increased the biomasses of Poaceae (+39.31%) and Asteraceae (+17.83%; Figure 1 and Supplementary Table S2). Drought significantly reduced the biomass of target species across all species (−23.23%), as well as within Amaranthaceae (−32.02%), Poaceae (−35.73%), and Asteraceae (−32.39%; Figure 1 and Supplementary Table S2). Furthermore, warming significantly increased the biomass of the native community across all target species classifications (all species: +23.72%; Amaranthaceae: +16.72%; Poaceae: +20.90%; Asteraceae: +31.84%), and post hoc pairwise comparisons revealed that at the level of all target species, the combined warming and drought treatment significantly increased the native community biomass compared with the drought treatment (+29.94%; Figure 1e and Supplementary Table S2). Drought significantly reduced native community biomass across all target species (all species: −27.86%; Amaranthaceae: −36.15%; Poaceae: −22.09%; Asteraceae: −24.05%).

**Figure 2:**
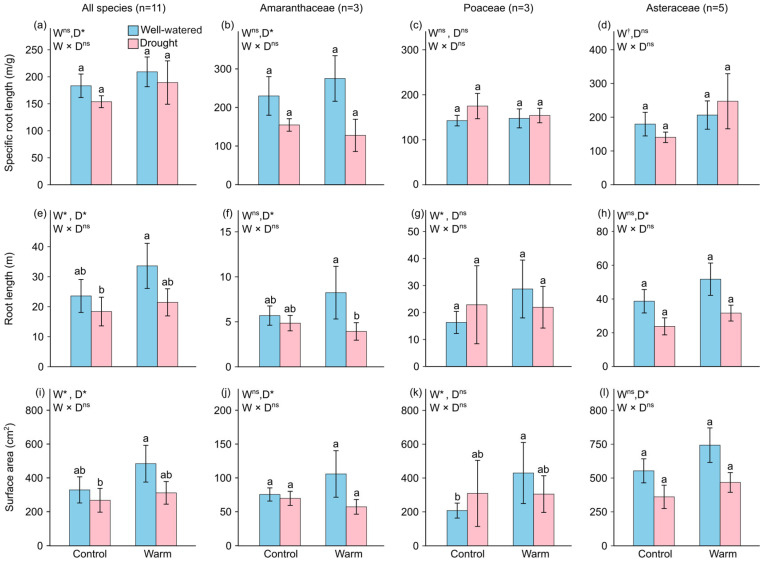
Modeled mean values (±SE) of the specific root length of the target species for (**a**) all species, (**b**) Amaranthaceae, (**c**) Poaceae, and (**d**) Asteraceae; the root length of the target species for (**e**) all species, (**f**) Amaranthaceae, (**g**) Poaceae, and (**h**) Asteraceae; and the root surface area of the target species for (**i**) all species, (**j**) Amaranthaceae, (**k**) Poaceae, and (**l**) Asteraceae under each combination of two warming (normal vs. warming) and two drought (well-watered vs. drought) treatments. Model terms (W, Warm; D, Drought) whose effects are significant (*p* < 0.05) are indicated with asterisks (*), and those whose effects are marginally significant (0.05 < *p* < 0.1) are indicated with daggers (†), ns indicates non-significant (*p* ≥ 0.1) Different lowercase letters above the bars indicate significant differences (*p* < 0.05) between treatment combinations based on post hoc pairwise comparisons of estimated marginal means (emmeans). The four treatment combinations consisted of two levels of warming (normal vs. warming) and two levels of drought (well-watered vs. drought).

Interestingly, we found a marginally significant interactive effect of warming and drought on the biomass of the target species within the Amaranthaceae (*p* = 0.0997; this suggestive result should be interpreted with caution; Figure 2g and Supplementary Table S2). Specifically, under normal conditions, drought increased the biomass proportion of Amaranthaceae species (+25.79%), whereas under the warming treatment, drought decreased the biomass proportion (−11.91%).

### 2.2. Root Functional Traits

On average, the warming treatment only increased the SRL of the target species within the Asteraceae family (+41.72%; Figure 2d and Supplementary Table S3), with no significant effect on the SRL in other taxonomic groups. Drought significantly reduced the SRL of all species (−12.59%; Figure 2a and Supplementary Table S3) and the Amaranthaceae family (−44.10%; Figure 2b and Supplementary Table S3). The warming treatment increased the root lengths of all species (+31.19%; Figure 2e) and the Poaceae family (+29.27%; Figure 2g and Supplementary Table S3). Drought significantly reduced the root length of all species (−30.35%; Figure 2e), as well as the Amaranthaceae (−36.94%; Figure 2f) and Asteraceae families (−38.69%; Figure 2h and Supplementary Table S3). Consistent with the results for root length, the warming treatment increased the root surface area of all species (+33.39%; Figure 2i) and the Poaceae family (+42.28%; Figure 2k and Supplementary Table S3). Drought significantly reduced the root surface area of all species (−28.79%; Figure 2e and Supplementary Table S3), as well as the Amaranthaceae (−29.94%; Figure 2f) and Asteraceae families (−36.12%; Figure 2h and Supplementary Table S3).

## 3. Discussion

We found that warming did not significantly increase the biomass of all species, yet significantly enhanced the biomass within Poaceae and Asteraceae, underscoring the importance of family level analysis. Pooling multi-species data may obscure differential responses among taxonomic groups, highlighting the necessity for conducting family level comparisons. The positive biomass response of Poaceae plants to warming may be attributed to their fibrous root systems. Fibrous roots are typically characterized by high SRL, high branching density, and shallow distribution, conferring a competitive advantage in heterogeneous resource environments [33,34]. This root architecture may enable invasive Poaceae species to efficiently capture nutrients mobilized by the warming-induced acceleration of soil organic matter decomposition, thereby translating resource pulses into growth advantages [29]. The positive response of invasive Asteraceae species to warming may benefit from their taproot systems, which facilitate access to deeper soil water, as well as the temperature sensitivity of allelochemical synthesis [30]. The three invasive Amaranthaceae species used in this study were all C4 plants whose photosynthetic systems are typically adapted to higher temperatures [31,32]. The warming treatment may not have exceeded the optimal temperature threshold, potentially explaining the absence of significant growth stimulation in this family.

Warming significantly increased the biomass of native communities across all target species groups, which is consistent with the general understanding that warming promotes plant growth [35,36,37]. Interestingly, post hoc comparisons revealed that the biomass of the native community under combined warming and drought was significantly higher than that under drought alone, suggesting that warming partially alleviated the suppressive effects of drought on the native community. This mitigating effect may occur through mechanisms such as enhanced soil nutrient mineralization. However, these benefits did not equally favor invasive plants, implying that the native community may possess a greater buffering capacity under resource fluctuations.

Drought significantly reduced the biomass of invasive plants across all families, which is consistent with the well-established growth-limiting effects of drought stress [15,17]. However, for biomass proportion, a metric reflecting competitive outcomes, Amaranthaceae exhibited a distinct response pattern. Under normal conditions, drought increased the biomass proportion of Amaranthaceae, indicating that this family could maintain or even enhance its competitive status relative to the native community under water-limited conditions. In contrast, under the combined warming and drought conditions, the proportion of Amaranthaceae biomass decreased, resulting in a marginally significant interactive effect. This finding suggests that Amaranthaceae may possess physiological mechanisms for drought adaptation, such as high water-use efficiency and osmotic adjustment capacity, which enables them to maintain their competitiveness under water-limited conditions [38]. However, when warming and drought co-occur, the intensified evaporative demand may exceed the tolerance threshold of Amaranthaceae, or their water acquisition strategies may conflict with carbon allocation under high temperatures, leading to reduced relative competitiveness. This nonlinear response provides direct evidence for the assertion that the combined effects of warming and drying may generate regulatory impacts distinct from those of single factors and reinforces the notion that studies examining single factors alone may either overestimate or underestimate invasion risks under compound stresses.

Root traits are key determinants of the plant capacity for water and nutrient acquisition, and their adjustments directly reflect plant adaptive strategies to environmental stress [29]. In our study, root trait responses to warming and drought exhibited clear family level differentiation, offering mechanistic insights into the sources of competitive advantage among different invasive plant groups. Warming significantly increased root length and root surface area in Poaceae but had no effect on SRL. This pattern suggests that Poaceae invasive species employ a root expansion strategy in response to warming to capture nutrients released by enhanced mineralization [39,40]. The increases in root length and surface area likely confer a greater resource acquisition capacity to invasive Poaceae species, which is consistent with their significant biomass enhancement. In contrast, drought primarily affected the root traits of Amaranthaceae and Asteraceae plants, but in markedly different directions. Invasive Amaranthaceae species exhibited significant reductions in SRL, root length, and root surface area under drought conditions, representing comprehensive root investment contraction. This contraction strategy may reflect the prioritization of limited carbon resources for root maintenance and survival rather than expansion under drought stress [41]. In contrast, Asteraceae displayed a differentiation strategy, whereas root length and root surface area significantly decreased under drought conditions and SRL showed an increasing trend. An increased SRL typically indicates that a plant can produce longer roots per unit biomass investment, representing an adaptive strategy to enhance resource acquisition efficiency [20,42]. Notably, although the invasive Amaranthaceae species increased their biomass proportion under drought conditions, their root traits contracted. A possible explanation is that, despite reducing absolute root investment under drought conditions, Amaranthaceae may enhance aboveground resource-use efficiency or competitive ability, thereby maintaining an advantage in the overall biomass proportion. This suggests that invasive plant performance under stress involves multidimensional trade-offs and that single traits alone may not fully predict competitive outcomes.

Our results indicate that the interactive effects of warming and drought were not significant for most of the measured parameters, indicating that the combined effects of warming and drought were primarily additive rather than interactive. This implies that warming and drought influence alien plant invasion through relatively independent physiological pathways. Under our experimental conditions, these factors have not yet produced strong synergistic or antagonistic interactions.

## 4. Materials and Methods

### 4.1. Study Species

**Figure 3:**
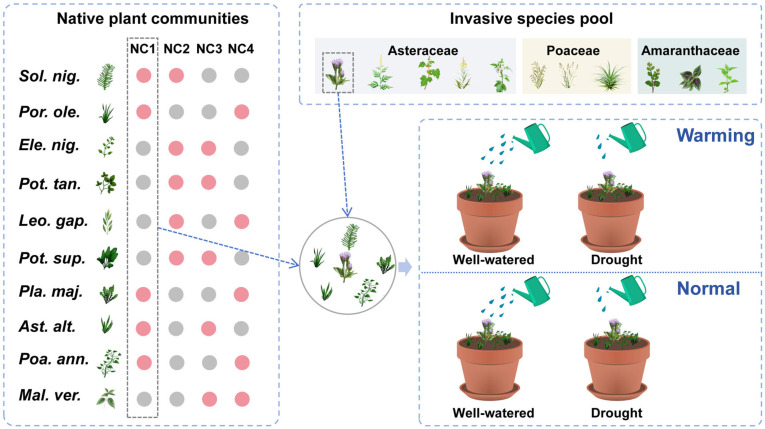
A schematic illustration of the experimental design. Each native community was assembled from five randomly selected species from a pool of 10 common native species, with each of the 10 species appearing exactly twice across the four communities. Pink dots indicate the species composition within the community.

We conducted a multispecies experiment to examine the effects of warming, drought, and their interaction on alien plant invasion (Figure 3). Eleven invasive plant species were selected as target species in our experiment, belonging to Amaranthaceae (three species), Poaceae (three species), and Asteraceae (five species). We also utilized 10 native species from 9 genera spanning 8 families (Supplementary Table S1). To determine whether each species was alien or native to China, we consulted The Checklist of the Alien Invasive Plants in China [43] and the Flora of China database (www.efloras.org, accessed on 1 December 2025).

We sowed both invasive and native species into round plastic trays (diameter × height: 18 cm × 3 cm) filled with sterilized commercial peat moss (Pindstrup Plus, Pindstrup Mosebrug A/S, Pindstrup, Denmark). Because preliminary observations indicated varying germination rates among the species, seeds were sown on different dates to ensure that all species reached a comparable growth stage prior to transplantation (Supplementary Table S1). The seeded trays were transferred to a greenhouse under LED lights, where relative humidity was maintained at 65% and temperature at approximately 24–30 °C.

### 4.2. Experimental Design

The experiment was conducted in a greenhouse at Lanzhou University (36.03° N, 103.40° E). On 11 December 2024, uniformly sized seedlings of each species were transplanted into 2.5 L circular plastic pots (top diameter: 18.5 cm; bottom diameter: 12.5 cm; height: 15 cm) filled with a 1:1 volume mixture of washed sand and fine vermiculite. To account for the natural diversity of native plant communities while ensuring balanced representation of each species, we employed a randomized assembly approach following [44]. The 10 native species were randomly combined into four distinct native communities, each consisting of five species. This design ensured that each of the 10 native species appeared exactly twice across the four communities, allowing us to simulate multiple native community compositions while maintaining equal statistical representation of all native species. Following the native plant community combination, each pot contained one invasive species planted in the center, with five native species evenly spaced in a square around it (Figure 3; Supplementary Table S1). The experiment employed a full factorial design, resulting in 176 pots: two levels of warming (normal vs. warming) × two levels of drought (well-watered vs. drought) × four native community replicates × 11 invasive species.

After transplantation, all pots were randomly assigned positions, and their positions were re-randomized every 15 days throughout the experiment. On 18 December 2024 (i.e., one week later), we began warm and drought treatments. To apply the warming treatment, each pot was enclosed in a transparent PVC cover (0.3 mm thickness and 43 cm height). In the normal treatment, a perforated PVC sheet was placed around the pot to eliminate warming effects (Supplementary Figure S1). During the experiment, the temperature in the warming treatment was elevated by 2–3 °C. For the well-watered treatment, each pot was watered every four days with 200 mL of water. For the drought treatment, each pot was watered every four days with 100 mL of water. To ensure adequate nutrients for normal plant growth, fertilization was applied every 15 days, and each pot received 100 mL of nutrient solution at a concentration of 2 g/L per application (Peters Professional 20–20–20 General Purpose Fertilizer, Everris, NA Inc., Dublin, OH, USA: 20% total N, 20% available PO4, 20% soluble potash). On days when fertilization coincided with the drought treatment, the watering volume for the drought treatment was reduced by 100 mL.

### 4.3. Plant Harvest

On 8 March 2025 (i.e., 87 days after transplanting), we harvested the aboveground and belowground biomasses of both the invasive plants and native communities. All the biomass samples were dried at 65 °C for 72 h and weighed. Based on the aboveground and belowground biomasses of the invasive species and native community, we calculated the total biomass production per pot (i.e., aboveground biomass + belowground biomass) and the biomass proportion of the target species in each pot (i.e., the total biomass of the target species/[the total biomass of the target species + the total biomass of the native community]), which represented the dominance of the target species in the communities, employing a methodology similar to previous studies [45]. In addition, we measured the root traits of the target species. Root systems were scanned using an EPSON Perfection V850 Pro scanner (Seiko Epson Corporation, Suwa, Japan), and root length and root surface area were analyzed using image analysis software (WinRHIZO 2020c Pro, Regent Instruments, Québec, QC, Canada). Root surface area was calculated as the sum of the lateral surface area of all root segments, assuming each segment as a cylinder (π × diameter × length). Specific root length (SRL) was then calculated as root length divided by belowground biomass of the target species.

### 4.4. Statistical Analyses

All statistical analyses were conducted in R version 4.1.3 [46] using linear mixed-effects models fitted with the lme function from the ‘nlme’ package [47]. Warm treatments (normal vs. warming), drought treatments (well-watered vs. drought), and their interactions were included as fixed effects to test their effects on plant performance. The response variables included the biomass of the target species, biomass of the native community, biomass proportion of the target species, SRL of the target species, root length of the target species, and root surface area of the target species. Data transformations were applied to the biomass of the target species and native communities, as well as the root length and root surface area of the target species, to meet the assumptions of normality and homoscedasticity.

Analyses including all species and family identities of the target species, as well as those of native communities in which they grew, were included as random effects. In separate analyses conducted within each family (Amaranthaceae, Poaceae, and Asteraceae), the invasive plant species and native community identity were included as random effects. Furthermore, we conducted post hoc pairwise comparisons of estimated marginal means (emmeans) across various treatment combinations, utilizing the ‘emmeans’ function in the R package ‘emmeans’ [48].

## 5. Study Limitations

Although our results demonstrated that the combined effects of warming and drought are primarily additive rather than interactive, several limitations should be noted. First, greenhouse experimental conditions cannot fully replicate the complexity of natural ecosystems, and factors such as soil microbial interactions, enemy regulation, and pulse effects of extreme climatic events were not incorporated. Second, we employed a fixed-volume watering regime to simulate drought conditions rather than gravimetric monitoring, which may not fully capture pot-to-pot variation in actual soil moisture loss due to differences in plant transpiration and evaporation. Third, the experiment covered only a single growing season, which was insufficient to reveal the cumulative effects of long-term warm–dry stress or plant adaptive evolutionary processes. Fourth, although this study encompassed 11 invasive species from three families, the limited number of species within each family may have affected the robustness of the family level conclusions owing to potential interspecific variation. We suggest that future research should combine field-based in situ warming and drought network experiments with long-term monitoring of competitive dynamics between invasive and native plants and integrate multidimensional indicators, such as the soil microbiome and plant chemical defense, to develop a more comprehensive mechanistic framework for understanding invasion under warm–dry scenarios.

## 6. Conclusions

Future warming–drying climates will exert taxon-specific effects on invasive plants, with different families exhibiting different root trait adjustment strategies in response to warming and drought stress. Poaceae invasive species gain a growth advantage under warming through root expansion; Amaranthaceae invasive species maintain relative competitiveness under drought, but this advantage may be weakened by combined warm–dry stress; and Asteraceae responds to drought by adjusting root efficiency and remaining relatively stable. The interactive effects of warming and drought were only marginally significant for the biomass proportion of invasive Amaranthaceae species, whereas additive effects were dominant for most other indicators. Our experiment revealed that, from a family level comparative perspective, the taxon-specific mechanisms by which warming–drying climates influence invasion processes provide a scientific basis for developing targeted early warning and management strategies.

## Data Availability

Should the manuscript be accepted, the data supporting the results will be archived in Dryad and the data DOI will be included at the end of the article.

## Supplementary Materials

The following supporting information can be downloaded at: https://www.mdpi.com/article/10.3390/plants15071018/s1, Table S1: Details of the study species used in the experiment; Table S2: Results of linear mixed- effects models testing the effects of warm (normal vs. warming), drought (well-watered vs. drought), and their possible interactions on the biomass of both the alien target species and the native community, as well as the biomass proportion of the alien target species in each pot. Significant effects (*p* < 0.05) are bold, and marginally significant effects (0.05 < *p* < 0.1) are underlined; Table S3: Results of linear mixed- effects models testing the effects of warm (normal vs. warming), drought (well-watered vs. drought), and their possible interactions on the specific root length of the alien target species, root length of the alien target species, as well as the surface area of the alien target species in each pot. Significant effects (*p* < 0.05) are bold, and marginally significant effects (0.05 < *p* < 0.1) are underlined; Figure S1: The overview of the greenhouse experiment and a comparison example of different treatments.
