# Supplementary material for: Responses of Invasive Plants from Different Families to Warming and Drought
Source: Plants (Basel). 2026 Mar 26;15(7):1018. doi: 10.3390/plants15071018 (PMC13074960; doi:10.3390/plants15071018)
Supplement: Supplementary file 1 [file plants-15-01018-s001.zip › plants-4209925-supplementary.pdf]

Supporting information

Responses of Invasive Plants from Different Families to Warming and Drought

Yu Zhang [1], Yu Tian [1,*] and Xiaochen Zhao [2]

[1] Key Laboratory of Tree and Grass Geneticsand Breeding, College of Forestry and Grassland Science, Jilin Agricultural University, Changchun 130118, China; zhangyu08240318@163.com
[2] Shenzhen Institute of Standards and Technology, Shenzhen 518033, China; zhaoxiaochen@sist.org.cn
* Correspondence: tianyu@jlau.edu.cn

Table S1 Details of the study species used in the experiment.

Species	Genus	Family	Status	Community	Sowing date
Amaranthus retroflexus	Amaranthus	Amaranthaceae	Invasive	/	11/25/2024
Amarapthus spinosus	Amaranthus	Amaranthaceae	Invasive	/	11/14/2024
Amaranthus hybridus	Amaranthus	Amaranthaceae	Invasive	/	11/14/2024
Cenchrus spinifex	Cenchrus	Poaceae	Invasive	/	12/7/2024
Avena fatua	Avena	Poaceae	Invasive	/	11/14/2024
Lolium perenne	Lolium	Poaceae	Invasive	/	12/2/2024
Ambrosia artemisiifolia	Ambrosia	Asteraceae	Invasive	/	11/14/2024
Bidens Pilosa	*Bidens*	Asteraceae	Invasive	/	11/18/2024
Erigeron canadensis	*Erigeron*	Asteraceae	Invasive	/	10/17/2024
Flaveria bidentis	*Flaveria*	Asteraceae	Invasive	/	11/14/2024
Xanthium italicum	*Xanthium*	Asteraceae	Invasive	/	12/2/2024
Oenothera biennis	*Oenothera*	Onagraceae	Invasive	/	11/14/2024
Datura stramonium	*Datura*	Solanaceae	Invasive	/	11/14/2024
Solanum nigrum	*Solanum*	Solanaceae	Native	NC1/NC2	11/14/2024
Portulaca oleracea	*Portulaca*	Portulacaceae	Native	NC1/NC4	12/2/2024
Eleusine indica	*Eleusine*	Poaceae	Native	NC2/NC3	12/7/2024
Potentilla tanacetifolia	*Potentilla*	Rosaceae	Native	NC2/NC3	11/14/2024
Leonurus japonicus	*Leonurus*	Labiatae	Native	NC2/NC4	11/14/2024
Potentilla supina	*Potentilla*	Rosaceae	Native	NC2/NC3	11/14/2024
Plantago major	*Plantago*	Plantaginaceae	Native	NC1/NC4	11/14/2024
Aster altaicus	*Aster*	Asteraceae	Native	NC1/NC3	11/22/2024
Poa annua	*Poa*	Poaceae	Native	NC1/NC4	11/25/2024
Malva verticillatavar	*Malva*	Malvaceae	Native	NC3/NC4	11/14/2024

Table S2 Results of linear mixed- effects models testing the effects of warm (normal vs. warming), drought (well-watered vs. drought), and their possible interactions on the biomass of both the alien target species and the native community, as well as the biomass proportion of the alien target species in each pot. Significant effects ($p < 0.05$) are bold, and marginally significant effects ($0.05 < p < 0.1$) are underlined.

	Biomass of the target species			Biomass of the native community			Biomass proportion of the target species		
**Factor**	***df***	χ^2	***P***	***df***	χ^2	***p***	***df***	χ^2	***P***
**All Species (n=11)**		(sqrt-transformed)			(sqrt-transformed)			(sqrt-transformed)	
Warm (W)	1	1.125	0.289	1	15.357	**0.0001**	1	0.019	0.890
Drought (D)	1	4.10	**0.025**	1	34.316	**<0.0001**	1	0.352	0.553
W × D	1	0.405	0.525	1	0.323	0.570	1	0.288	0.592
Random effects		***SD***			***SD***			***SD***	
Family		0.304			0.136			0.112	
Species		0.419			0.278			0.127	
Native community		0.065			0.133			0.042	
Residual		0.383			0.381			0.119	
R^2 of the model	R_m^2		R_c^2		R_m^2	R_c^2		R_m^2	R_c^2
	0.068		0.673		0.150	0.523		0.001	0.382
**Amaranthaceae (n=3)**		(sqrt-transformed)			(sqrt-transformed)			/	
Warm (W)	1	1.650	0.199	1	5.690	**0.017**	1	0.003	0.957
Drought (D)	1	7.539	**0.006**	1	37.766	**<0.0001**	1	0.063	0.801
W × D	1	1.626	0.202	1	0.180	0.672	1	2.711	0.0997
Random effects		***SD***			***SD***			***SD***	
Species		0.457			0.219			0.161	
Native community		0.172			0.179			0.079	
Residual		0.244			0.255			0.086	
R^2 of the model	R_m^2		R_c^2		R_m^2	R_c^2		R_m^2	R_c^2
	0.049		0.810		0.378	0.718		0.011	0.814
**Poaceae (n=3)**		(sqrt-transformed)			(sqrt-transformed)			(sqrt-transformed)	
Warm (W)	1	5.681	**0.017**	1	3.001	0.083	1	0.210	0.647
Drought (D)	1	7.427	**0.006**	1	4.170	**0.041**	1	0.160	0.690
W × D	1	0.132	0.717	1	0.018	0.894	1	0.052	0.820
Random effects		***SD***			***SD***			***SD***	
Species		0.102			0.210			0.030	
Native community		<0.0001			<0.0001			<0.0001	
Residual		0.380			0.463			0.120	
R^2 of the model	R_m^2		R_c^2		R_m^2	R_c^2		R_m^2	R_c^2
	0.223		0.275		0.110	0.262		0.009	0.0653
**Asteraceae (n=5)**		(sqrt-transformed)			(natural-log-transformed)			(asin-transformed)	
Warm (W)	1	2.745	0.098	1	7.210	**0.007**	1	<0.0001	0.981
Drought (D)	1	10.4551	**0.001**	1	11.223	**0.001**	1	0.689	0.407
W × D	1	0.345	0.557	1	0.318	0.573	1	0.118	0.732
Random effects		***SD***			***SD***			***SD***	
Species		0.491			0.330			0.138	
Native community		<0.0001			0.199			0.048	
Residual		0.446			0.372			0.131	
R^2 of the model	R_m^2		R_c^2		R_m^2	R_c^2		R_m^2	R_c^2
	0.083		0.585		0.121	0.577		0.003	0.556

R_m^2 represents marginal R^2, R_c^2 represents conditional R^2.

Table S3 Results of linear mixed- effects models testing the effects of warm (normal vs. warming), drought (well-watered vs. drought), and their possible interactions on the specific root length of the alien target species, root length of the alien target species, as well as the surface area of the alien target species in each pot. Significant effects (p < 0.05) are bold, and marginally significant effects (0.05 < p < 0.1) are underlined.

	Specific root length			Root length			Surface area		
**Factor**	***df***	χ^2	***P***	***df***	χ^2	***p***	***df***	χ^2	***P***
**All Species (n=11)**	(natural-log-transformed)			(natural-log-transformed)			(natural-log-transformed)		
Warm (W)	1	1.603	0.206	1	5.474	**0.020**	1	5.96	**0.015**
Drought (D)	1	3.99	**0.046**	1	6.960	**0.008**	1	4.975	**0.026**
W × D	1	0.010	0.921	1	0.998	0.318	1	1.228	0.268
Random effects		***SD***			***SD***			***SD***	
Family		<0.0001			0.840			0.871	
Species		0.187			0.415			0.388	
Native community		0.117			0.265			0.186	
Residual		0.615			0.870			0.874	
R^2 of the model	R_m^2		R_c^2		R_m^2	R_c^2		R_m^2	R_c^2
	0.031		0.141		0.036	0.572		0.033	0.567
**Amaranthaceae (n=3)**	(natural-log-transformed)			(natural-log-transformed)			(natural-log-transformed)		
Warm (W)	1	0.027	0.870	1	0.045	0.833	1	0.006	0.936
Drought (D)	1	6.717	**0.010**	1	8.492	**0.004**	1	5.283	**0.022**
W × D	1	0.328	0.567	1	0.911	0.340	1	0.547	0.460
Random effects		***SD***			***SD***			***SD***	
Species		0.298			0.409			0.321	
Native community		0.164			0.435			0.453	
Residual		0.716			0.558			0.571	
R^2 of the model	R_m^2		R_c^2		R_m^2	R_c^2		R_m^2	R_c^2
	0.118		0.281		0.094	0.577		0.063	0.518
**Poaceae (n=3)**	(natural-log-transformed)			(natural-log-transformed)			(natural-log-transformed)		
Warm (W)	1	0.002	0.962	1	5.438	**0.020**	1	6.419	**0.011**
Drought (D)	1	1.128	0.288	1	0.069	0.792	1	0.160	0.690
W × D	1	0.886	0.347	1	1.713	0.191	1	1.978	0.160
Random effects		***SD***			***SD***			***SD***	
Species		0.171			0.475			0.493	
Native community		<0.0001			0.250			0.090	
Residual		0.371			0.791			0.757	
R^2 of the model	R_m^2		R_c^2		R_m^2	R_c^2		R_m^2	R_c^2
	0.036		0.204		0.104	0.386		0.124	0.391
**Asteraceae (n=5)**	(natural-log-transformed)			(sqrt-transformed)			(sqrt-transformed)		
Warm (W)	1	0.002	0.093	1	2.282	0.131	1	2.500	0.114
Drought (D)	1	1.128	0.286	1	7.36	**0.007**	1	5.519	**0.019**
W × D	1	0.472	0.493	1	0.001	0.971	1	0.041	0.839
Random effects		***SD***			***SD***			***SD***	
Species		0.201			0.385			0.136	
Native community		0.055			0.286			0.250	
Residual		0.654			1.027			1.033	
R^2 of the model	R_m^2		R_c^2		R_m^2	R_c^2		R_m^2	R_c^2
	0.054		0.141		0.064	0.232		0.059	0.202

R_m^2 represents marginal R^2, R_c^2 represents conditional R^2.

Figure S1

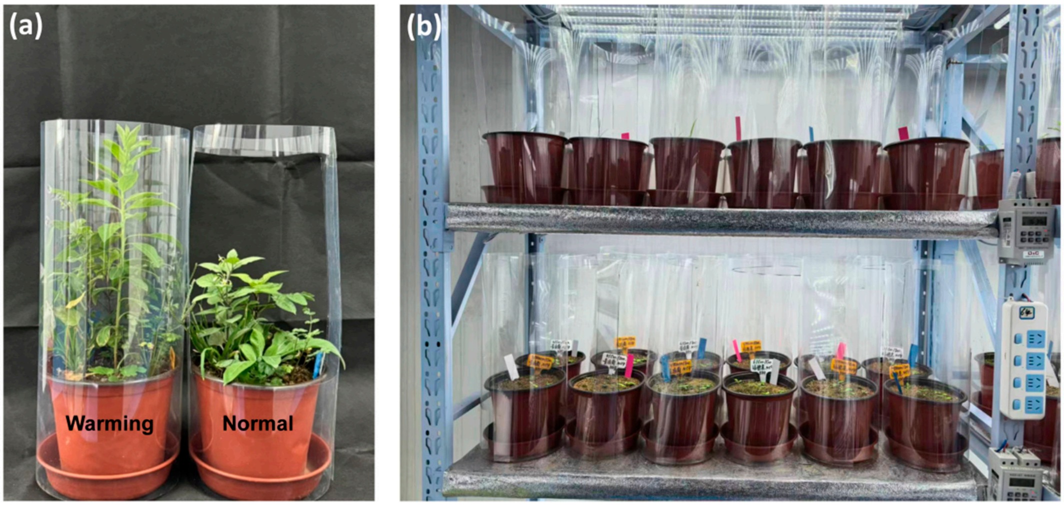

Figure S1 The overview of the greenhouse experiment and a comparison example of different treatments.
